# Stephen Lane Wood

**DOI:** 10.3897/zookeys.56.514

**Published:** 2010-09-17

**Authors:** Donald E. Bright

**Affiliations:** Department of Bioagricultural Sciences and Pest Management, C. P. Gillette, Museum of Arthropod Diversity, Colorado State University, Fort Collins, Colorado, 80527

Stephen Lane Wood was born in Providence, Utah, on July 2, 1924. From an early age, he had a keen interest in nature and wildlife. At age 14, Steve went to Nevada to visit his brother-in-law, T. O. (Ted) Thatcher, a specialist in scolytid systematics, who sent him into the woods to begin an insect collection for a class project. Steve dug his pocketknife into a pinhole in an aspen tree and eased out a small beetle, Trypodendron retusum (LeConte). In Steve’s words, “The attraction was immediate and permanent.” His interest in natural history lead him to attend Utah State University where he received his B. S. degree in 1946 and M. S. degree in 1948, majoring in Entomology. His Masters thesis was a survey of the Scolytidae of Logan Canyon in Utah and their host plants (publication #1). He received his Ph.D. in 1953 from the University of Kansas with a dissertation on a taxonomic revision of the North American Cryphalini (publication #4). During his graduate studies Steve met and interacted with the leaders in the field of bark beetle systematics, which highlighted his burgeoning career (Fig. 1).

**Figure 1.**
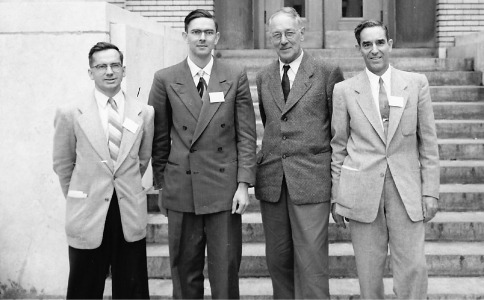
International Congress of Entomology, Montreal, Quebec. Left to right:  Boyd Thomas, Canadian Forestry Service, worked on larval Scolytidae; Steve Wood; Karl Schedl, Austria, world authority on Scolytidae; W. H. (Bill) Anderson, Washington D. C., worked on Scolytidae at U. S. National Museum, Smithsonian Institution.

After graduation, Steve joined the staff of the Canadian National Collection of Insects, Ottawa, Ontario, and remained there for three years until family considerations and the call of the Utah mountains inspired him to change employment. In 1956, he accepted the position of Assistant Professor in the Department of Zoology and Entomology at Brigham Young University in Provo, Utah and remained there until his death. After retirement, Steve continued his association and his research at BYU at the Monte L. Bean Life Sciences Museum as Professor Emeritus and Curator of Coleoptera Emeritus until ill health forced him to terminate his activities in 2008.

Steve served on a number of university committees and professional assignments. He was editor of the Great Basin Naturalist for many years and created the Great Basin Naturalist Memoirs series. He was a visiting Research Professor of Entomology at universities in San Jose, Costa Rica and Merida, Venezuela. He was the principal faculty planner in the development of the Monte L. Bean Life Science Museum on the BYU campus and was the Curator of the Coleoptera collection in the Life Science Museum until his retirement (Fig. 2).

**Figure 2.**
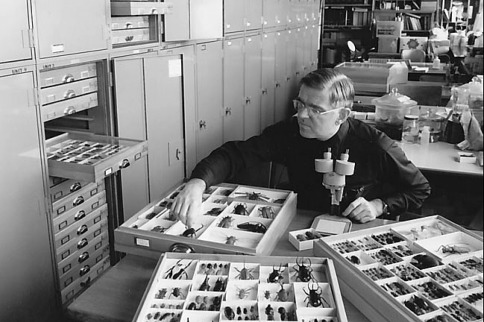
1978, Steve working on BYU collection, obviously not on bark beetles!

Steve authored or co-authored 109 publications on scolytid and platypodid systematics and described and named over 1000 species as new to science. His collection, containing many thousands of specimens, was housed in 300 large museum drawers in the Monte L. Bean Life Science Museum. He donated his collection to the Smithsonian Institution in Washington D. C. In April and early May, 2009, the collection was consolidated into 160 drawers and was transported to the Smithsonian. Steve also built a library of thousands of published articles pertaining to scolytid systematics and biology which was donated to the Smithsonian Institution along with the specimen collection.

During his career, Steve assisted with, or was involved with, several large projects. Probably the most important were a study of the bark and ambrosia beetles of North and Central America, which culminated in the publication of a large monograph in 1982, and a study of the bark and ambrosia beetles of South America, which culminated in the publishing of another large monograph in 2007. The 1982 monograph mentioned above is the standard taxonomic reference for these important forest insects and will likely continue so for decades to come. Likewise, the 2007 South American monograph will likely not be repeated for decades. Other projects included a study of the Scolytidae of Sri Lanka, which Steve expanded into a study of the Scolytidae of India, sponsored by the Smithsonian Institution and the Ceylon National Museum and a study of the Scolytidae of China, supported by a travel grant from the U. S. National Academy. This latter grant was used to bring Dr. Yin Hui-fen from Beijing to BYU. Neither of these latter two studies were completed and published due to financial constraints but preliminary manuscripts for both were prepared.

In 1981, I contacted Steve and suggested that we unite our efforts and compile a catalog of the world species of Scolytidae and Platypodidae. This collaboration resulted in the publication, in 1987, of a bibliography containing references to over 25,000 research articles, and in 1992, of a complete catalog of the Scolytidae and Platypodidae of the world.

Throughout his career, Steve was actively involved in collecting and observing bark beetle habits. He collected in at least 19 foreign countries, most Canadian Provinces, 25 states in Mexico, and in all contiguous states in the US except Washington and Vermont (Fig. 3). In 1972, he spent two weeks in New Guinea and two weeks in Australia and, in1976, he spent two weeks in India and six weeks in Sri Lanka collecting beetles for the Smithsonian Institution. He collected in Venezuela, Colombia, Finland, Central America, Japan and Russia (Fig. 4). His publications are replete with personal observations on the gallery pattern, site attacked, host plant, behavior, and other biological observations of the species treated.

**Figure 3.**
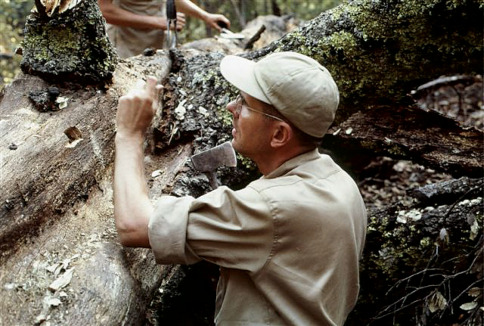
Dr. Stephen Wood collecting scolytines in Mexico. Photo courtesy of Ladd Livingston who, as an undergraduate student, accompanied Steve in Mexico.

**Figure 4.**
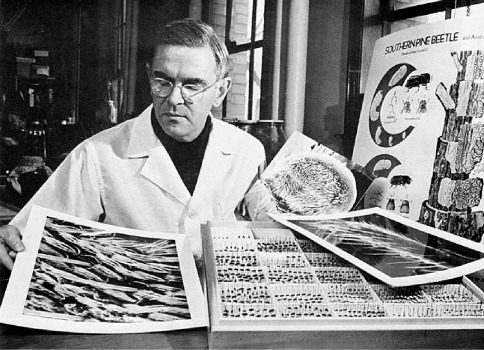
1978, Steve examining photo enlargements of bark beetle parts. On the desk is a tray of beetles he has collected from all areas of the world.

Steve passed away on July 1, 2009, at the age of 84 in Provo, Utah of age-related causes. His influence on the systematics of the bark and ambrosia beetles of the world is immeasurable and will be felt for decades to come.

## Publications of S. L. Wood

109) Wood SL (2007) Bark and Ambrosia beetles of South America (Coleoptera: Scolytidae). Monte L. Bean Life Science Museum, Brigham Young University. 900 pp.

108) Wood SL (1993) Revision of the genera of Platypodidae (Coleoptera). Great Basin Naturalist 53: 259–281.

107) Wood SL, Bright  DE (1992) A catalog of the of the Scolytidae and Platypodidae (Coleoptera). Part 2: Taxonomic Index, Volumes A & B. Memoirs of the Great Basin Naturalist 13. 1553 pp.

106) Wood SL (1992) Nomenclatural changes and new species of Platypodidae and Scolytidae (Coleoptera). Great Basin Naturalist 52: 78–88.

105) Wood SL (1992) Nomenclatural changes in Scolytidae and Platypodidae (Coleoptera). Great Basin Naturalist 52: 89–92.

104) Wood SL, Stevens GC, Lezama HJ (1992) Los Scolytidae (Coleoptera) de Costa Rica. Clave de la subfamilia Scolytinae, Tribu: Corthylini. Revista de Biologia Tropical 40: 247–286.

103) Wood SL, Stevens GC, Lezama HJ (1991) Los Scolytidae de Costa Rica: clave de géneros y de la subfamilia Hylesinae (Coleoptera). Revista de Biologia Tropical 39: 125–148.

102) Wood SL, Stevens GC, Lezama HJ (1991) Scolytidae (Coleoptera) de Costa Rica II. Clave para la subfamilia Scolytinae, tribus: Scolytini, Ctenophorini, Micracini, Ipini, Dryocoetini, Xyleborini y Cryphalini. Revista de Biologia Tropical 39: 279–306.

101) Wood SL (1989) Nomenclatural changes and new species of Scolytidae (Coleoptera), Part IV. Great Basin Naturalist 49: 167–185.

100) Wood SL (1988) Recent advances in knowledge of the distribution and classification of the Scolytidae (Coleoptera). Proceedings of the International Congress of Entomology 18: 410.

99) Wood SL (1988) Systematic position of the Scolytidae and Platypodidae (Coleoptera). Proceedings of the International Congress of Entomology 18: 40.

98) Wood SL (1988) Nomenclatural changes and new species of Scolytidae (Coleoptera), Part III. Great Basin Naturalist 48: 188–195.

97) Wood SL (1988) Nomenclatural changes and new species of Scolytidae (Coleoptera). Great Basin Naturalist 48: 31–38.

96) Wood SL (1987) Six new Scolytidae (Coleoptera) from Mexico. Great Basin Naturalist 47: 547–550.

95) Wood SL, Bright DE (1987) A Catalog of the of the Scolytidae and Platypodidae (Coleoptera). Part 1. Bibliography. Memoirs of the Great Basin Naturalist 11. 685 pp.

94) Wood SL (1986) A reclassification of the genera of Scolytidae (Coleoptera). Memoirs of the Great Basin Naturalist 9. 126 pp.

93) Wood SL, Huang FS (1986) New genus of Scolytidae (Coleoptera) from Asia. Great Basin Naturalist 46: 465–467.

92) Wood SL (1986) New synonymy and new species of American bark beetles (Coleoptera: Scolytidae), Part XI. Great Basin Naturalist 46: 265–273.

91) Wood SL (1986) New Pseudoxylechinus (Coleoptera: Scolytidae) from India. Great Basin Naturalist 43: 468.

90) Wood SL, Huang F (1986) New genus of Scolytidae (Coleoptera) from Asia. Great Basin Naturalist 46: 465–467.

89) Wood SL, Yin F (1986) Relict occurrence of three “American” Scolytidae (Coleoptera) in Asia. Great Basin Naturalist 46: 461–464.

88) Wood SL (1985) New synonymy and new species of bark beetles (Coleoptera: Scolytidae). Great Basin Naturalist 45: 266–275.

87) Wood SL (1984) New generic synonymy and new genera of Scolytidae (Coleoptera). Great Basin Naturalist 44: 223–230.

86) Wood SL (1984) Hypocryphalus mangiferae (Stebbing, 1914) (Insecta, Coleoptera): proposed conservation under the plenary powers. Bulletin of Zoological Nomenclature 41: 189–190.

85) Wood SL (1984) Review of: J. B. Mitton and K. B. Sturgeon (Eds), Bark beetles of North American conifers. New York Entomological Society Journal 92: 93–94.

84) Wood SL (1984) New synonymy and new species of American bark beetles (Coleoptera: Scolytidae), Part X. Great Basin Naturalist 43: 113–119.

83) Wood SL (1983) New synonymy and new species of American bark beetles (Coleoptera: Scolytidae), Part IX. Great Basin Naturalist 43: 647–659.

82) Wood SL (1983) Scolytodes auratus panamensis (Escarabajito de Guarumo, Cecropia Petiole Borer). In: Jansen DH (Ed) Costa Rican NaturalHistory. University of Chicago Press, 758–759.

81) Wood SL (1982) New species of American bark beetles (Coleoptera: Scolytidae). Great Basin Naturalist 42: 223–231.

80) Wood SL (1982) The bark and ambrosia beetles of North and Central America (Coleoptera: Scolytidae), a taxonomic monograph. Memoirs of the Great Basin Naturalist 6. 1359 pp.

79) Wood SL (1981) Nomenclatural changes and new species in P1atypodidae and Scolytidae (Coleoptera). Great Basin Naturalist 41: 121–128.

78) Wood SL (1980) Los Scolytidae de Mexico. In: Premer simposio nacional sobre parasitologia forestal, 18–19 de Febrero de 1980, Uruapan, Michoacan, Mexico. Memoria Sociedad Mexicana de Emtomologia, 13–57.

77) Wood SL (1980) New American bark beetles (Coleoptera: Scolytidae), with two recently introduced species. Great Basin Naturalist 40: 353–358.

76) Wood SL (1980) New genera and new generic synonymy in Scolytidae (Coleoptera). Great Basin Naturalist 40: 89–97.

75) Wood SL (1979) New synonymy and new species of American bark beetles (Coleoptera: Scolytidae), Part VIII. Great Basin Naturalist 39: 133–142.

74) Wood SL (1979) A catalog of the Coleoptera of America north of Mexico: Family Platypodidae. U.S. Department of. Agriculture, Agriculture Handbook 529–141. 5 pp.

73) Wood SL (1978) New synonymy and new species of American bark beetles (Coleoptera: Scolytidae), Part VII. Great Basin Naturalist 38: 397–405.

72) Wood SL (1978) A reclassification of the subfamilies and tribes of Scolytidae (Coleoptera). Annales of the Societe Entomologique de France 14: 95–122.

71) Wood SL (1977) New synonymy and new species of American bark beetles (Coleoptera: Scolytidae), Part VI. Great Basin Naturalist 37: 511–522.

70) Wood SL (1977) New synonymy and new species of American bark beetles (Coleoptera: Scolytidae), Part V. Great Basin Naturalist 37: 383–394.

69) Wood SL (1977) New synonymy and new species of American bark beetles (Coleoptera: Scolytidae), Part IV. Great Basin Naturalist 37: 207–220.

68) Wood SL (1977) Introduced and exported American Scolytidae (Coleoptera). Great Basin Naturalist 37: 57–74.

67) Wood SL (1976) New synonymy and new species of American bark beetles (Coleoptera: Scolytidae), Part III. Great Basin Naturalist 35: 347–355.

66) Wood SL (1976) Reply to comments on the proposal to conserve Liparthrum Wollaston, 1864 (Coleoptera: Scolytidae). Bulletin of Zoological Nomenclature 33: 4pp.

65) Wood SL (1975) Proposed conservation under the plenary powers of the name Phloeotribus Latreille, 1804 (Insecta: Coleoptera, Scolytidae). Bulletin of Zoological Nomenclature 32: 122–123.

64) Wood SL (1975) New synonymy and new species of American bark beetles (Coleoptera: Scolytidae, Part II. Great Basin Naturalist 35: 391–401.

63) Wood SL (1975) New synonymy and new species of American bark beetles (Coleoptera: Scolytidae). Great Basin Naturalist 35: 21–32.

62) Wood SL (1974) New synonymy and records of American bark beetles (Coleoptera: Scolytidae). Great Basin Naturalist 34: 277–290.

61) Wood SL (1974) Proposed conservation under the plenary powers of the name Dryocoetes Eichhoff, 1864 (Insecta: Coleoptera, Scolytidae). Bulletin of Zoological Nomenclature 31: 232–233.

60) Wood SL (1974) Proposed conservation under the plenary powers of the name Xyleborus Eichhoff, 1864 (Insecta: Coleoptera, Scolytidae). Bulletin of Zoological Nomenclature 31: 230–231.

59) Wood SL (1974) Proposed conservation under the plenary powers of the name Phloeosinus Chapuis, 1869 (Insecta: Coleoptera, Scolytidae). Bulletin of Zoological Nomenclature 31: 236–237.

58) Wood SL (1974) Proposed conservation under the plenary powers of the name Liparthrum Wollaston, 1864 (Insecta: Coleoptera, Scolytidae). Bulletin of Zoological Nomenclature 31: 234–235.

57) Wood SL (1974) Proposed conservation under the plenary powers of the name Phloeotribus Latreille, 1804 (Insecta: Coleoptera, Scolytidae). Bulletin of Zoological Nomenclature 31: 122–123.

56) Wood SL (1974) New species of American Corthylus (Coleoptera: Scolytidae). Great Basin Naturalist 34: 181–202.

55) Wood SL (1974) New species of American Corthylini (Coleoptera: Scolytidae). Great Basin Naturalist 34: 135–150.

54) Wood SL (1974) New species of American bark beetles (Coleoptera: Scolytidae). Brigham Young University Science Bulletin, Biological Series 19. 67 pp.

53) Wood SL (1973) New species of American Microcorthylus (Coleoptera: Scolytidae). Great Basin Naturalist 33: 265–275.

52) Wood SL (1973) New synonymy in American bark beetles (Scolytidae: Coleoptera). Part III. Great Basin Naturalist 33: 169–188.

51) Wood SL (1973) On the taxonomic status of Platypodidae and Scolytidae (Coleoptera). Great Basin Naturalist 33: 77–90.

50) Wood SL (1973) A correction in the taxonomic identity of Platypus parallelus (Fabricius) (Coleoptera: Platypodidae). The Coleopterists Bulletin 27: 51–52.

49) Wood SL (1972) Review of K. E. Schedl, Monographie der familie Platypodidae Coleoptera. Science 178: 1085–1086.

48) Wood SL (1972) Notes on the classification of the tribe Scolytini (Coleoptera: Scolytidae). Bulletin of Entomological Researeh 62: 243–246.

47) Wood SL (1972) New synonymy in the bark beetle tribe Cryphalini (Coleoptera: Scolytidae). Great Basin Naturalist 32: 40–54.

46) Wood SL (1972) New synonymy in American bark beetles (Scolytidae: Coleoptera). Part II. Great Basin Naturalist 32: 190–201.

45) Wood SL (1972) New synonymy in American bark beetles (Scolytidae: Coleoptera). Great Basin Naturalist 31: 140–152.

44) Wood SL (1972) Family Scolytidae (Ipidae). In: M. H. Hatch, The beetles of the Pacific Northwest, Part 5. University of Washington, Publications in Biology 16, 395–428.

43) Wood SL (1972) New records and species of American Platypodidae (Coleoptera). Great Basin Naturalist 31: 243–253.

42) Wood SL (1972) New species of bark beetles (Scolytidae: Coleoptera) from western North America. Great Basin Naturalist 31: 69–76.

41) Wood SL (1971) New records and species of neotropical bark beetles (Scolytidae: Coleoptera). Part V. Brigham Young University Science Bulletin, Biological Series 15(3). 54 pp.

40) Wood SL (1969) New synonymy and records of Platypodidae and Scolytidae (Coleoptera). Great Basin Naturalist 29: 113–128.

39) Wood SL (1969) Additions to the horned bark beetle genus Cactopinus Schwarz (Scolytidae). The Coleopterists Bulletin 23: 42–51.

38) Wood SL (1969) New records and species of neotropical bark beetles (Scolytidae: Coleoptera). Part IV. Brigham Young University Science Bulletin, Biological Series 10(2). 46 pp.

37) Wood SL (1968) A key to the species of the genus Cnesinus LeConte (Coleoptera: Scolytidae) of North and Central America. Great Basin Naturalist 28: 88–110.

36) Wood SL (1968) New records and species of neotropical bark beetles (Scolytidae: Coleoptera). Part III. Great Basin Naturalist 28: 1–15.

35) Wood SL (1967) New records and species of neotropical bark beetles (Scolytidae: Coleoptera). Part II. Great Basin Naturalist. 27: 119–141.

34) Wood SL (1967) New records and species of neotropical bark beetles (Scolytidae: Coleoptera). Great Basin Naturalist 27: 79–97.

33) Wood SL (1967) New species of bark beetles (Coleoptera: Scolytidae), mostly Mexican. Part VII. Great Basin Naturalist 27: 37–57.

32) Wood SL (1967) Cryphalus Erichson, 1836 (Insecta: Coleoptera): proposed designation of a type-species under the plenary powers. Bulletin of Zoological Nomenclature 24: 121–122.

31) Wood SL (1966) New records and species of neotropical Platypodidae (Coleoptera). Great Basin Naturalist 26: 45–70.

30) Wood SL (1966) New synonymy in the Platypodidae and Scolytidae (Coleoptera). Great Basin Naturalist 26: 17–33.

29) Wood SL (1965) The genus Eupagiocerus Blandford (Scolytidae: Coleoptera). Great Basin Naturalist 25: 31–35.

28) Wood SL (1964) New species of North American Pityophthorus Eichhoff (Coleoptera: Scolytidae). Great Basin Naturalist 24: 59–70.

27) Wood SL (1963) A revision of the bark beetle genus Dendroctonus Erichson (Coleoptera: Scolytidae). Great Basin Naturalist 23: 1–117.

26) Wood SL (1962) Miscellaneous taxonomic notes on Scolytidae (Coleoptera). Great Basin Naturalist 22: 76–82.

25) Wood SL (1961) New species of bark beetles (Coleoptera: Scolytidae) mostly Mexican, Part VI. Great Basin Naturalist 21: 87–107.

24) Wood SL (1961) A new Dactylipalpus (Coleoptera: Scolytidae) from the Philippine Islands. Great Basin Naturalist 21: 8–9.

23) Wood SL (1961) New records and species of Scolytidae (Coleoptera) from Colombia. Great Basin Naturalist 21: 1–7.

22) Wood SL (1961) An alternate proposal to the suggested validation of Myelophilus Eichhoff, 1878. (Insecta: Coleoptera). Bulletin of Zoological Nomenclature 18(5): 319–321.

21) Wood SL (1961) A key to the North American genera of Scolytidae. The Coleopterists Bulletin 15: 41–48.

20) Wood SL (1960) New records and species of Scolytidae (Coleoptera) from western North America. Great Basin Naturalist 20: 59–69.

19) Wood SL (1960) Coleoptera: Platypodidae and Scolytidae. Insects of Micronesia 18(1): 1–73.

18) Wood SL (1959) New records and species of Arizona bark beetles (Coleoptera: Scolytidae). Great Basin Naturalist 19: 57–62.

17) Wood SL (1959) New species of bark beetles (Coleoptera: Scolytidae), mostly Mexican, Part V. Great Basin Naturalist 19: 1–7.

16) Wood SL (1958) Bark Beetles of the genus Pityoborus Blackman (Coleoptera: Scolytidae). Great Basin Naturalist 18: 46–56.

15) Wood SL (1958) Some virtually unknown North American Platypodidae (Coleoptera). Great Basin Naturalist 18: 37–40.

14) Wood SL (1957) New species of bark beetles (Coleoptera: Scolytidae), mostly Mexican, Part IV. Great Basin Naturalist 17: 105–110.

13) Wood SL (1957) A new generic name for and some biological data on an unusual Central American beetle (Coleoptera: Platypodidae). Great Basin Naturalist 17: 103–104.

12) Wood SL (1957) Results from the Danish expedition to the French Cameroons 1949–50. XXIII. Coleoptera: Platypodidae and Scolytidae. Bulletin Institut francis d’Afrique Noire 19: 1272–1273.

11) Wood SL (1957) Distributional notes on and synonymies of some North American Scolytidae (Coleoptera). The Canadian Entomologist 89: 396–403.

10) Wood SL (1957) Ambrosia beetles of the tribe Xyloterini (Coleoptera: Scolytidae) in North America. The Canadian Entomologist 89: 337–354.

9) Wood SL (1957) The North American allies of Hylobius piceus (De Geer) (Coleoptera: Curculionidae). The Canadian Entomologist 89: 37–43.

8) Wood SL (1956) New species of bark beetles (Coleoptera: Scolytidae), mostly Mexican, Part III. The Canadian Entomologist 88: 247–258.

7) Wood SL (1956) New species of bark beetles (Coleoptera: Scolytidae), mostly Mexican, Part II. The Canadian Entomologist 88: 231–240.

6) Wood SL (1956) New species of bark beetles (Coleoptera: Scolytidae), mostly Mexican, Part I. The Canadian Entomologist 88: 141–154.

5) Wood SL (1954) Bark beetles of the genus Carphoborus Eichhoff (Coleoptera: Scolytidae) in North America. The Canadian Entomologist 86: 502–526

4) Wood SL (1954) A revision of North American Cryphalini (Scolytidae: Coleoptera). University of Kansas Science Bulletin 36: 959–1090.

3) Wood SL (1952) Observations of the homologies of the copulatory apparatus in male Coleoptera. Annals of the Entomological Society of America 45: 613–617.

2) Wood SL (1951) Two new species and a new genus of Scolytidae (Coleoptera) from Utah. Journal of the Kansas Entomological Society 24: 31–32.

1) Wood SL (1951) The Scolytidae of the Logan Canyon area of Utah and their host plants. Utah Academy of Sciences, Arts and Letters 26: 127–128.

